# Association between work-related biomechanical risk factors and the occurrence of carpal tunnel syndrome: an overview of systematic reviews and a meta-analysis of current research

**DOI:** 10.1186/s12891-015-0685-0

**Published:** 2015-09-01

**Authors:** Agnessa Kozak, Grita Schedlbauer, Tanja Wirth, Ulrike Euler, Claudia Westermann, Albert Nienhaus

**Affiliations:** Institute for Health Services Research in Dermatology and Nursing (CVcare), University Medical Center Hamburg-Eppendorf, Hamburg, Germany; Institution for Statutory Accident Insurance and Prevention in the Health and Welfare Services, Hamburg, Germany; Institute and Policlinic for Occupational and Social Medicine (IPAS), Technical University Dresden, Dresden, Germany

## Abstract

**Background:**

Occupational risks for carpal tunnel syndrome (CTS) have been examined in various occupations, and several systematic reviews (SRs) have been published on this topic. There has been no critical appraisal or synthesis of the evidence in the SRs. The aims of this study are (1) to synthesise the observational evidence and evaluate the methodological quality of SRs that assess the effect of biomechanical risk factors on the development of CTS in workers, (2) to provide an update of current primary research on this association, (3) to assess a potential dose-response relationship.

**Methods:**

We searched MEDLINE, EMBASE, CINAHL, the Cochrane Library and the reference lists of articles. The first step covered SRs (1998–2014), and the second step covered current primary studies (2011–2014). The methodological quality of the SRs was evaluated by using the AMSTAR-R tool; primary studies were assessed using a list of 20 items. A qualitative approach was used for synthesising evidence. In addition, we undertook a meta-analysis of the primary studies to determine risk ratios in the dose-response relationship.

**Results:**

We identified ten SRs that covered a total of 143 original studies. Seven primary studies met the criteria for inclusion, of which four provided longitudinal data. We found high quality of evidence for risk factors such as repetition, force and combined exposures. Moderate quality of evidence was observed for vibration, and low quality of evidence was found for wrist postures. An association between computer use and CTS could not be established. Recent primary studies supported the existence of a significant relationship between CTS and repetition, force and combined exposure. The meta-analysis of current research revealed a dose-response relationship between CTS and the American Conference of Governmental Industrial Hygienists’ (ACGIH) threshold limit value (TLV) for hand-activity level (HAL). Those between the action limit and TLV and above TLV had RR of 1.5 (95 % CI 1.02–2.31) and RR 2.0 (95 % CI 1.46–2.82), respectively.

**Conclusions:**

Occupational biomechanical factors play a substantial role in the causation of CTS. Data from current primary studies on dose-response suggest that the risk of CTS increases with the ACGIH TLV levels.

**Electronic supplementary material:**

The online version of this article (doi:10.1186/s12891-015-0685-0) contains supplementary material, which is available to authorized users.

## Background

Carpal tunnel syndrome (CTS) is a pathophysiological peripheral mononeuropathy, caused by an increase in the tissue pressure in the carpal tunnel. This leads to pressure damage of the N. medianus, linked to sensory and motor failures in the affected area. CTS is the most frequent compression syndrome of a peripheral nerve. A review of occupational populations showed that the prevalence of CTS varies greatly with the diagnostic criteria, population and study type, and it may range from 0.6 to 61 % [1, 2]. In population-based studies, the prevalence rates range from 1 to 6 % [3–6]. CTS mainly affects women and increases with age. In Swedish and Italian population studies, the annual incidence for women was 428 and 506 per 100,000 respectively. This is about three times greater than the corresponding values for men, namely 182 and 139 cases per 100,000 respectively [7, 8]. The causes of CTS may be local (e.g., cysts), regional (e.g., rheumatoid arthritis) or systemic (e.g., diabetes) [9]. There is increasing scientific evidence that development of CTS is promoted by highly repetitive manual tasks, involving awkward hand/wrist postures, with flexion and extension of the hands, forceful exertion or hand/arm vibration during work [1, 10]. Some occupational groups are more exposed than others, due to the nature of their work. These are mostly occupations requiring the frequent use of hand-held vibratory tools and high levels of physical exposure, particularly during assembly work, food processing and packaging [11]. As CTS is common in the general population and is multi-causal, it is legitimate to ask to what extent it is caused by occupational factors. This has been a controversial issue for many years [12–16]. For example, Thurston [17] argued that occupational factors — such as repetition, vibration or force — are not the primary cause of CTS and that it was more likely that these activities trigger symptoms or exacerbate existing latent symptoms. In a prospective study on the aetiology of CTS in the industrial sector, the authors found out that individual factors, such as age, being overweight, gender, hand anthropometrics and hand dominance play a much greater role in causing CTS than occupational factors, such as force, repetition, duration of employment and type of employment [18–20]. However, early systematic reviews (SRs) concluded that there is sufficient evidence for an association between occupational exposure to biomechanical factors and the development of CTS [1, 10, 21, 22].

In recent years, several SRs and meta-analyses have been published on the aetiology of CTS in the occupational context. There has however been no critical evaluation of the SRs or a discussion of the results. The “overview of systematic reviews” represents a new approach to synthesising evidence from several SRs [23]. Overviews can potentially provide a broad summary of empirical research on a specific issue [24]. The information provided by these overviews is essentially dependent on the validity of the primary studies and the SRs that they include [25]. As SRs may very rapidly become dated, it is advisable to include the most recent publications, too [26, 27].

This overview aims to synthesise and critically evaluate the quality of SRs and current primary studies assessing the relationship between occupational biomechanical factors and CTS in working populations. Another objective is to quantify the dose-response relationship using the American Conference of Governmental Industrial Hygienists (ACGIH) threshold limit value (TLV) for hand-activity level (HAL) model.

## Methods

The literature search and analysis took place in two steps. The first step consisted of an explicit search for SRs. The procedure was based on the methods paper published by the Clearinghouse of Systematic Reviews of the Partnership for European Research in Occupational Safety and Health (PEROSH) [28]. In the second step, primary studies were identified and evaluated. This study was conducted according to the Meta-analysis of Observational Studies in Epidemiology (MOOSE) checklist (see Additional file 1) [29].

### Search strategy and study selection

An electronic literature search for SRs was performed in the MEDLINE (via Pubmed), EMBASE (via Ovid), CINAHL (via EBSCO) and COCHRANE databases. It covered the publication period from 1998 to 2014 (last update 27.7.14) and used predefined search strings and terms. In order to identify aetiological studies in the occupational context, we employed the sensitive search string developed by Mattioli et al. [30], in combination with the terms for exposure (exposure; physical load; risk factor*; repetiti*; hand-arm vibration; force), outcome (carpal tunnel syndrome; median nerve neuropathy; median nerve entrapment; nerve compression syndrome) and study design (meta-analysis; review; not letter, editorial, comment). The search strategy is listed in the Additional file 2. We also searched for additional sources within the references of relevant publications. The following inclusion and exclusion criteria for SRs were applied:

- Population: employed adults.
- Exposure: biomechanical factors in the occupational context (exclusion: studies on diagnostic testing, treatment or rehabilitation).
- Outcome: CTS as primary outcome (exclusion: CTS as concomitant disease, e.g., in diabetes mellitus).
- Design: SRs and meta-analyses (exclusion: narrative reviews, editorials, commentaries).

To update the analysis, we conducted a primary literature search using MEDLINE, EMBASE and CINAHL databases. The same sensitive search string was employed, except for the partial string for SRs and meta-analyses. The last comprehensive literature search was performed in the meta-analysis published by Spahn et al. [31]. Our search therefore included the period January 2011 to 2014 (last update 31.8.2014). The following inclusion and exclusion criteria were applied for primary studies:

- Population: employed adults.
- Exposure: consideration of at least one biomechanical exposure factor, giving degrees of association or raw data.
- Outcome: conservative CTS case definition: (a) abnormal findings in the nerve conduction study (NCS) that indicated dysfunction of the N. medianus in the carpal tunnel and (b) either clinical signs (a positive Phalen’s or Tinel’s sign) or symptoms indicative of CTS such as paraesthesia, numbness or pain.
- Design: peer review article with case control, cross-sectional and cohort studies.

Six languages (English, German, Italian, Spanish, Portuguese and Russian) were considered. The studies were selected independently by two reviewers (AK, TW). In the event of disagreement, consensus was achieved by discussion. When no consensus could be achieved, a third reviewer (GS) was consulted. Data were extracted by one reviewer (AK). To verify accuracy of extraction, a second and a third reviewer (TW, GS) checked all relevant data for each included SR and primary study. Data extracted from the studies is listed in the Additional file 3.

### Degree of overlap between the SRs

If primary studies are included in more than a single SR on the same research question, this can lead to bias in the interpretation of the results of the overview. For this reason, it was necessary for the overview to determine the extent to which the primary studies overlapped in the different SRs. This is presented in Table 1. Additionally, a calculation was performed of the percentage of primary studies included in more than one SR. A measure of overlap was also calculated — the “Corrected Covered Area” (CCA), using the method proposed by Pieper et al. [26]. The included primary studies were extracted from each SR, documented and calculated in an Excel table (SR x primary studies). CCA can be interpreted as the overlap area of studies that occur at least twice in SRs, after correction for the first time each primary study was counted (index publications). The frequency of repeated occurrence of index publications in SRs (N) is divided by the product of index publications (r) and reviews (c), minus by the number of index publications (r; see calculation formula). CCA values between 0 and 5 indicate slight overlap; values between 6 and 10 moderate overlap, values between 11 and 15 high overlap and values above 15 very high overlap [26].

**Table 1:** Overlap of original research studies included in the systematic reviews

Author, year	1	2	3	4	5	6	7	8	9	10
1. Abbas et al. 1998 [21]	**17**	0	9	1	7	10	9	8	3	0
2. Sulsky et al. 2005 [44]		**34**	12	3	12	14	6	13	0	3
3. Palmer et al. 2007 [22]			**38**	5	19	18	16	19	4	2
4. Thomsen et al. 2008 [45]				**9**	4	4	1	3	1	3
5. Lozano-Calderón et al. 2008^a^ [46]					**66**	18	12	16	5	2
6. van Rijn et al. 2009 [35]						**44**	21	21	3	3
7. Barcenilla et al. 2012 [41]							**37**	22	3	0
8. Spahn et al. 2012^a^ [31]								**55**	2	1
9. You et al. 2014 [43]									**8**	0
10. Mediouni et al. 2014 [42]										**6**

Bold numbers are studies included in each SRs

^a^Included primary studies that were used for the analysis of occupational risk factors, but which were not listed explicitly, e.g., in the form of an evidence table. Consequently all studies from all tables, figures or text were extracted when they were used for the analysis of occupational factors. This was used to determine overlap

$$ CCA = \frac{N-r}{rc-r} $$

***N*** is the number of publications included (with duplicate counts) in the evidence synthesis of individual SRs; ***r*** is the number of index publications (individual primary studies) and c the number of SRs.

### Quality assessment

The validity of the included SRs was critically and independently assessed by two reviewers using the Assessment of Multiple Systematic Reviews – Revised (AMSTAR-R), an instrument that was specifically developed to assess the methodological quality of SRs [32]. Between 11 and 44 points could be reached on the AMSTAR-R score. To differentiate between the SRs, the numerical score was converted to quality grades: *A* = 37–44 (very good); *B* = 29–36 (good); *C* = 21–28 (moderate); *D* = 13–20 (poor) points [33]. The inter-rater reliability between two reviewers was determined with Cohen’s kappa coefficient [34].

The evaluation of the validity of the primary studies was based on the criteria developed by van Rijn et al. [35] and Ariëns et al. [36] (see Additional file 4). These were adapted to suit the research question and then summarised to a cumulative score with a maximum of 20 points. Quality was rated as methodologically high (≥14 points), moderate (8 to 13 points) or poor (≤7 points).

### Quality of evidence

Due to the heterogeneity of the primary studies and the overlap of the study pool of the SRs included, no formal evidence synthesis was possible with the Grades of Recommendation, Assessment, Development and Evaluation (GRADE) approach [37]. We therefore determined the quality of evidence using a qualitative approach for each type of occupational exposure. The assessment of the quality of evidence depended on the methodological validity of the SRs (AMSTAR-R score), together with the consistency of the results between the SRs (direction of effect and significance) [38, 39]. We gave greater weight to recently published SRs; older SRs provided supportive evidence [27]. The following classification was specified:

- High – consistent evidence in very good SRs (at least one grade A review).
- Moderate – consistent evidence in good SRs (at least one grade B review).
- Low – one SR of moderate quality (at least grade C) and significant results and/or good SRs (grade B), with some inconsistent results.
- Poor – none of the above conditions were met (i.e., consistent findings in low-quality SRs (grade D), or inconsistent findings in multiple SRs).

The results of the primary studies served to support the assessment of the quality of evidence, as both their methodological validity and their consistency were considered; i.e., when at least two valid primary studies (≥14 points) gave consistent results, the quality of the evidence from the SRs was upgraded.

### Statistical analyses

Comparable primary studies were pooled in the form of quantitative data synthesis and presented as forest plots. The relative risk (RR) was calculated and 95 % confidence intervals (CI) were generated. The heterogeneity of individual studies was quantified using the Chi-square (*χ*^*2*^) and *I*^*2*^ statistics. If there was statistically significant heterogeneity (*χ*^*2*^, *p* <0.10 and *I*^*2*^ > 50 %), then the pooled effect estimate was determined with the random effects model. Otherwise, a fixed effects model was used [40]. The analyses were applied to current primary studies and were conducted using RevMan Version 5.2.

### Ethics

Ethical approval was not required as the study focused only on analysing secondary literature without any involvement of human subjects, tissues or medical records.

## Results

### SRs and meta-analyses

**Fig. 1:**
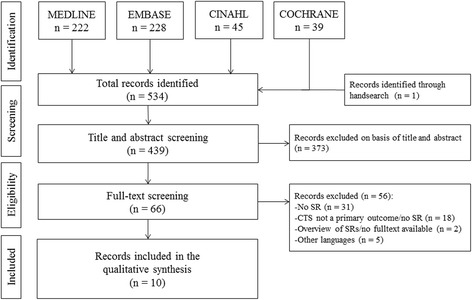
Flowchart of the selected systematic reviews

A total of ten relevant SRs were included. The flow diagram (Fig. 1) shows the selection of SRs identified by the electronic and hand search. The number of primary studies per SR varied from 6 to 66. Taken together, the ten SRs covered a total of 143 primary studies (index publications); these were cited up to 314 times in the SRs. 35 % of the index publications were cited in two to three SRs and about 29 % in four to six SRs (Table 1). The CCA value was 13.3, which indicates a high degree of overlap. Table 2 shows the detailed characteristics of the included SRs. In half of the SRs, a meta-analysis was performed [21, 31, 41–43]. Five of the other SRs presented the results qualitatively in the form of an evidence table [22, 35, 44–46]. Two SRs concentrated exclusively on the link between computer use and CTS [42, 45]. A meta-analysis by You et al. [43] only examined the link between non-neutral wrist postures and CTS. The paper by Sulsky et al. [44] is a report from the Occupational Insurance Association for Safety at Work; this was not published as a peer review article.

**Table 2:** Study characteristics of the included systematic reviews and meta-analyses

Author, year	Analysis	AMSTAR-R grade	Country	Years included	No. of studies included	Study designs	A priori quality criteria	The study’s aim was to …
You et al. 2014 [43]	MA	C	US	1980–2012	*n* = 8	CC = 2; CS = 6	Recognition of bias by sensitivity analysis	... conduct a meta-analysis of existing studies to evaluate the evidence of the relationship between wrist posture at work and CTS
Mediouni et al. 2014 [42]	MA	B	FR	1992–2012	*n* = 6	C = 2; CS = 4	Strengths and limitations acknowledged	... conduct a systematic review and meta-analysis of the available epidemiological data on the association between computer work exposure and CTS
Barcenilla et al. 2012 [41]	MA	B	AU	1980–2009	*n* = 37	C = 3; CC = 5; CS = 28	Risk of Bias Tool	... examine the association between workplace exposure and CTS by meta-analysis, with respect to exposure to hand force, repetition, vibration and wrist posture
Spahn et al. 2012 (in German) [31]	MA	C	DE	≤2011	*n* = 55	n/a	n/a	... conduct a systematic review and meta-analysis to identify associated and risk factors for CTS in the occupational setting
Van Rijn et al. 2009 [35]	SR	B	NL	1966–2007	*n* = 44	C = 5; CC = 9; CS = 30	16-item score	... provide a quantitative assessment of the exposure-response relationship between work-related physical and psychosocial factors and the occurrence of CTS in occupational populations
Lozano-Calderón et al. 2008 [46]	SR	D	US	≤2008	*n* = 51^a^; *n* = 33^b^; (total = 66)	C = 7^a^; CC = 12^a^; C = 29^a^; Other = 3^a^	Bradford Hill criteria for causation	... evaluate the quality and strength of scientific evidence supporting an aetiological relationship between a disease and a proposed risk factor, using a scoring system based on the Bradford Hill criteria for causal association – example of CTS
Thomsen et al. 2008 [45]	SR	C	DK	≤2004	*n* = 8	C = 4; CC = 2; CS = 2	Selected criteria (4 main domains)	... conduct a systematic review to examine evidence for an association between computer work and CTS
Palmer et al. 2007 [22]	SR	C	GB	≤2004	*n* = 38	n/a	n/a	... conduct a systematic review to assess occupational risk factors for CTS
Sulsky et al. 2005 [44]	SR	C	DE	1997–2003	*n* = 34	C = 10; CC = 2; CS = 22	Selected criteria (6 main domains)	... clarify the relationship between CTS and occupation using quality based criteria from the epidemiological literature
Abbas et al. 1998 [21]	MA	D	US	1980–1995	*n* = 17	C = 3; CC = 4; CS = 10	n/a	... conduct a meta-analysis on work-related CTS and to identify risk estimates and possible biases influencing the risk estimates

*Abbreviations: AMSTAR-R* Assessment of Multiple Systematic Reviews – Revised (numeric quality score in grades: A = 37–44; B = 29–36; C = 21-28; D = 13–20 points), *C* Cohort, *CC* Case control, *CS* Cross-sectional, *CTS* Carpal tunnel syndrome, *MA* Meta-analysis, *SR* Systematic review

^a^Studies investigating occupational factors alone

^b^Studies investigating both biological and occupational factors

Using AMSTAR-R scoring, three SRs were categorised as “grade B” [35, 41, 42], five as “grade C” [22, 31, 43–45] and two as “grade D” publications [21, 46]. With a single exception, the inter-rater reliability was good to very good (kappa: 0.38–0.87) (see Additional file 5).

SRs used different instruments and methods to assess the methodological quality of the included studies. The Cochrane Collaboration’s tool for assessing risk of bias was used by only a single meta-analysis [41]. Selective criteria were used in three additional studies, which all considered aspects such as study design, allocation of participants, outcome and exposure assessment, as well as the control of potential confounders [35, 44, 45]. You et al. [43] identified possible bias with sensitivity analyses; Mediouni et al. [42] provided the strengths and limitations of the original studies in an evidence table. Lozano-Calderón et al. [46] developed an assessment scheme in accordance with the Bradford-Hill criteria for causality and used this score to determine the quality and the strength of the evidence for the aetiological link between generally accepted risk factors for CTS (biological, occupational, as well as biological and occupational together).

The results from the SRs and meta-analyses are predominantly based on cross-sectional and case control studies; prospective longitudinal studies were in the minority. Table 3 shows the main results from the SRs.

**Table 3:** Main results of the included systematic reviews and meta-analyses stratified by the exposure factors

Author, year, ↓quality	Vibration (95 % CI)	Repetition (95 % CI)	Force (95 % CI)	Combined exposure (repetition and force) (95 % CI)	Wrist posture (95 % CI)	Computer exposure (95 % CI)
Barcenilla et al. 2012 [41] Grade B	NIOSH CTS def.: OR 2.7 (1.9–3.9); *n* = 12 studies Conservative CTS def.^a^: OR 5.4 (3.1–9.3); *n* = 3/3 (100 %) studies^d^	NIOSH CTS def.: OR 2.3 (1.8–3.0); *n* = 25 studies Conservative CTS def.^a^: OR 2.3 (1.7–2.9); *n* = 5/11 (45 %) studies^d^	NIOSH CTS def.: OR 2.2 (1.5–3.3); *n* = 13 studies Conservative CTS def.^a^: OR 4.2 (1.5–11.7); *n* = 3/5 (60 %) studies^d^	NIOSH CTS def.: OR 2.0 (1.4–2.9); *n* = 4/9 (44 %) studies^d^ Conservative CTS def.^a^: OR 1.9 (1.0–3.5); *n* = 5 studies	NIOSH CTS def.: OR 2.7 (1.3–5.5); *n* = 7 studies Conservative CTS def.^a^: OR 4.7 (0.4–53.3); *n* = 1/3 (33 %) studies^d^	/
Mediouni et al. 2014 [42] Grade B	/	/	/	/	/	Computer use: OR 1.7 (0.8-3.6); *n* = 5 studies; Keyboard/mouse use: OR 1.1 (0.6–2.0); OR 1.9 (0.9–4.2)
Van Rijn et al. 2009 [35] Grade B	OR 2.5–4.8; *n* = 3/5 (60 %) studies^d^	OR 0.5–9.4; *n* = 5/8 (62 %) studies^d^	OR 2.1–9.0; *n* = 3/7 (43 %) studies^d^	OR 3.2–8.4; *n* = 3/4 (80 %) studies^d^	OR 1.3–8.7; *n* = 4/5 (80 %) studies^d^	OR 2.1–4.4; *n* = 2/7 (29 %) studies^d^
You et al. 2014 [43] Grade C	/	/	/	/	Non-neutral wrist postures: RR 2.0 (1.7–2.4); *n* = 4/8 (50 %) studies^d^	/
Spahn et al. 2012 [31] Grade C	OR 2.6 (1.7–4.0); *n* = 6/9 (67 %) studies^d^	OR 2.7 (1.8–3.9); *n* = 11/13 (85 %) studies^d^ OR 2.1 (0.4–11.8); *n* = 3 cohort studies	OR 4.4 (1.4–13.6); *n* = 4/4 (100 %) studies^d^	OR 8.4 (7.8–8.9)^b^; *n* = 2/2 (100 %) studies^d^ OR 1.8 (1.4–2.2)^b^; *n* = 2/3 (67 %) cohort studies^d^	Flexion: OR 1.7 (1.0–2.6); *n* = 2/5 (40 %) studies^d^	Computer use: OR 1.8 (0.8–4.1); *n* = n/a studies
Sulsky et al. 2005 [44]^c^ Grade C	Insufficient evidence; *n* = 1 study	Consistent small positive association; *n* = 6 studies	Weak positive association of questionable validity; *n* = 3 studies	/	Insufficient evidence; *n* = 1 study	Insufficient evidence; *n* = 2 studies
Thomsen et al. 2008 [45] Grade C	/	/	/	/	/	Inconsistent evidence: OR < 1; *n* = 1 studies; OR > 1; *n* = 3 studies and *n* = 4 studies with no effect calculation or n.s.
Palmer et al. 2007 [22] Grade C	≥2 OR elevated risk (e.g., exposure ≥8 years); *n* = 7 studies	≥2 OR elevated risk (e.g., exposure <10 s. cycle time); *n* = 5 studies	Elevated risk for high-force jobs and activities (e.g., exposure >4 kg); *n* = n/a studies	Elevated risk for jobs with combined exposure; *n* = 1 study	≥2 OR elevated risk (e.g., exposure >17 or 20 h/week); *n* = 4 studies	Inconsistent results; *n* = 4 studies
Abbas et al. 1998 [21] Grade D	/	Significant predictor	Significant predictor	/	/	/
Lozano-Calderón et al. 2008 [46] Grade D	Ø OR 5.5; qBHs 6.3/21 points (range 5–8); *n* = 14/20 (70 %) studies^d^	Ø OR 4.0; qBHs 6.5/21 points (range 5–10); *n* = 30/45 (67 %) studies^d^	Ø OR n/a; qBHs 4.5/21 points (range 3–6); *n* = 15/31 (48 %) studies^d^	/	Flexion: Ø OR n/a; qBHs 5.4/21 points (range 4–8); *n* = 7/17 (41 %) studies^d^ Extension: Ø OR n/a; qBHs 3.6/21 points (range 3–4); *n* = 3/7 (43 %) studies^d^	/

*Abbreviations: CI* Confidence interval, *CTS* Carpal tunnel syndrome, *NIOSH* National Institute for Occupational Health and Safety (USA), *n.s.* not significant, *OR* Odds ratio, *RR* Relative risk, *qBHs* Quantitative score based on Bradford-Hill criteria (max. 21 points)

^a^Conservative CTS case definition: abnormal nerve conduction findings and symptoms (e. g., paraesthesia, pain, numbness) or clinical signs (positive Phalen’s sign or Tinel’s sign)

^b^Results refer to American Conference of Governmental Industrial Hygienist (ACGIH) Threshold Limit Value (TLV) for Hand-Activity Level (HAL)

^c^Results refer to eleven studies of high quality with minimised risk of bias

^d^Positive correlation observed

### Current primary studies

**Fig. 2:**
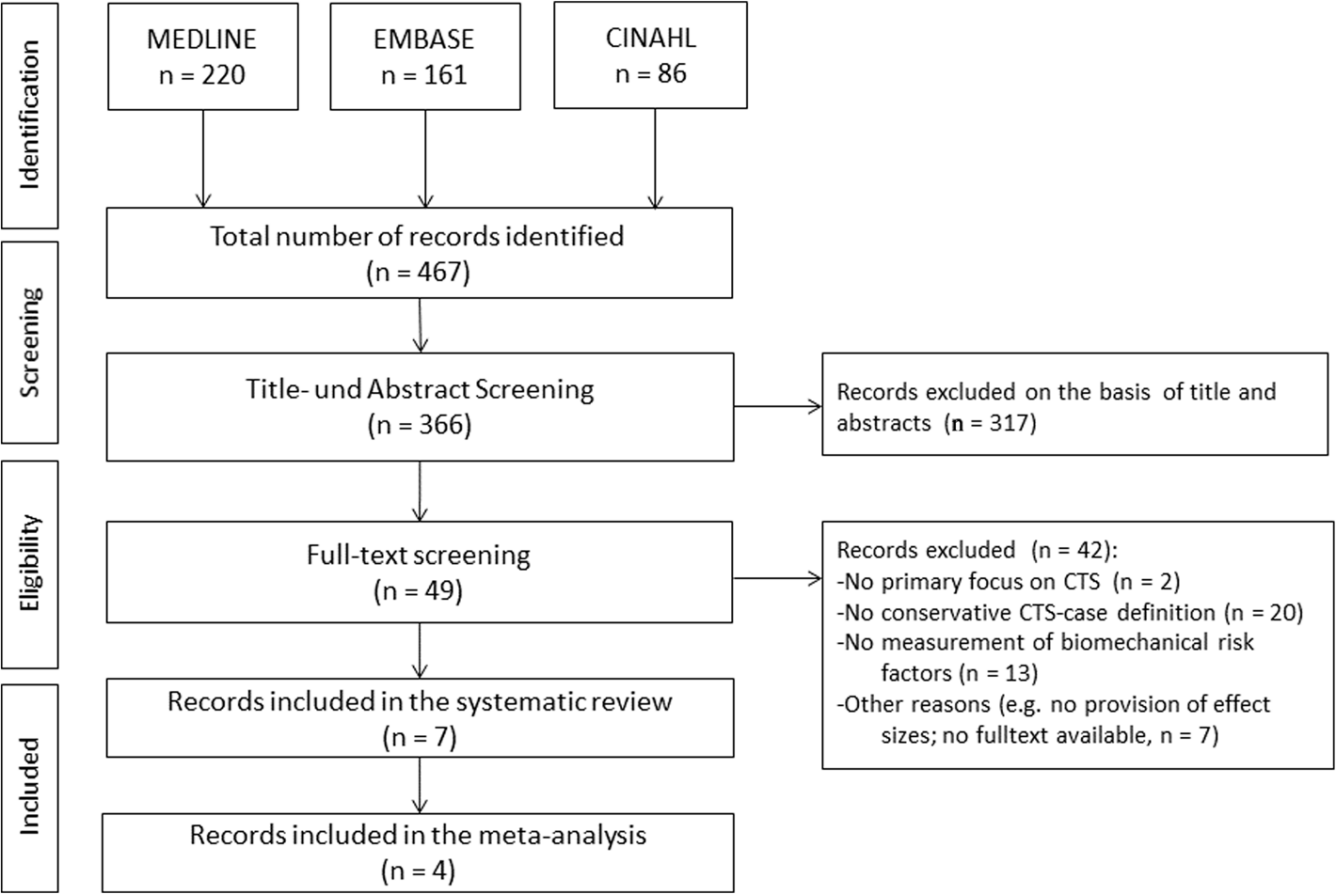
Flowchart of the selected primary studies

The selection of the primary studies employed the same selection process as for the SRs. After scrutinising 366 titles and abstracts, we reviewed 49 full texts and included a total of seven studies in the evidence synthesis (Fig. 2). The main reasons for exclusion were no conservative CTS definition (*n* = 20) or no investigation of biomechanical risk factors (*n* = 13). Of the included studies, four were of high quality and had a prospective design [47–50], although one publication only presented the baseline results [48]. The other three studies were of moderate quality, including one prospective study [51] and two case control studies (see Additional file 4) [52, 53]. In four studies, the exposures were measured with objective methods [47–50]. In two studies, exposures were self-reported [52, 53] and in one study, exposures were assessed with Job Exposure Matrices (JEM, US O*NET Database) [51]. A summary of the characteristics of the included primary studies along with the main results are shown in Table 4. All four studies of high quality determined the ACGIH TLV for HAL and were incorporated in the meta-analysis to clarify the dose-response relationship [47–50]. This score includes the combined exposure from peak force (PF) and repetition (HAL). HAL is based on frequency of exertion and duty cycle of exertion. PF is based on the peak effort exerted by the hand during the regular duty cycle. PF and HAL are combined into a single measure by calculating the ratio PF/(10-HAL). As proposed by ACGIH the TLV for HAL score <0.56 is considered below the Action Limit (AL) and is a category for general controls due to low risk. A score >0.78 is considered above the TLV and indicates a high risk. Scores between AL and TLV are considered to be possibly dangerous borderline exposures [54]. For the results of the meta-analysis see the paragraph on combined exposures.

**Table 4:** Study characteristics and main results of the included primary studies

Author, year	Design	Country	Study population	Outcome	Exposition	Main results from multivariate analyses (95 % Confidence Interval)	Confounder	Quality
Burt et al. 2011 [48]	CS (baseline)	US	*n* = 464 workers from hospital service^a^, engine and bus plant workplaces	Abnormal NCS + symptoms (hand diagram)	ACGIH TLV for HAL; Exertion/min. or time in %)^b^; Peak force (% time)^c^; Flexion/Extension (% time)^d^; Vibration (observed yes/no)	Peak force ≥20 % vs. <20 %: OR 1.3 (0.6–3.0); peak force ≥70 % vs. <20 %: OR 2.7 (1.3–5.7); exertion ≥15/min vs. <10/min if BMI ≥30: OR 3.4 (1.1–9.9); perceived exertion (unit increase): OR 1.14 (1.0-1.3); ≥AL- < TLV vs. <AL: OR 2.3 (0.6-8.9); ≥TLV vs. <AL: OR 3.0 (1.5–5.8); HAL (unit increase) if male: OR 1.4 (1.1–1.8)	Sex; BMI ≥ 30; arthritis; high blood pressure	High (16/20)
Burt et al. 2013 [47]	Cohort (2-years)	US	*n* = 347 workers from hospital service^a^, engine and bus plant workplaces	Abnormal NCS + symptoms (hand diagram)	ACGIH TLV for HAL; TLR; Exertion/min. or time in %)^b^; Peak force (% time)^c^; Flexion/Extension (% time)^d^; Vibration (observed yes/no)	Exertion/min. ≥20 % vs <20 %: HR 2.8 (1.2-6.8); exertion/min. ≥60 % vs. <20 %: HR 19.6 (6.0–64.2); TLR (unit increase): HR 1.4 (1.1-1.8)	BMI ≥ 30; job strain	High (19/20)
Garg et al. 2012 [49]	Cohort (6-years)	US	*n* = 551 workers from processing, assembly, manufacturing workplaces	Abnormal NCS + symptoms (intensity ≥25 %/d + duration ≥2 month)	ACGIH TLV for HAL; SI score	≥AL- < TLV vs. <AL: HR 1.4 (0.6-3.8); ≥TLV vs. <AL: HR 2.0 (0.8-5.0); SI score >6.1 vs. <6.1: HR 2.5 (1.0-6.1);	Age; BMI ≥ 30; gardening; depression; co-morbidity (other MSDs; arthritis)	High (18/20)
Evanoff et al. 2014 [51]	Cohort (3-years)	US	*n* = 1107 newly hired workers from construction, technical, laboratory, clerical and hospital service workplaces	Abnormal NCS + symptoms (hand diagram)	Job title based exposure on data from O*NET (job title and requirements): repetitive motion (5-point); static/ dynamic strength (7-point)	Results for most recent jobs (≤6 months): repetitive motion: OR 3.3 (1.4–7.8); static strength: OR 4.4 (1.4–13.9); dynamic strength: OR 3.6 (1.04-12.4);	Age; sex; BMI	Moderate (13/20)
Bonfiglioli et al. 2013 [50]	Cohort (2-years)	IT	*n* = 2194 workers from factories producing domestic appliances and nursery school	Abnormal NCS + symptoms (hand diagram)	ACGIH TLV for HAL; vibration (observed yes/no)	≥AL- < TLV vs. <AL: IRR 2.0 (1.2–3.2); ≥TLV vs. <AL: IRR 2.7 (1.5–4.9); HAL (unit increase): IRR 1.4 (1.2–1.6); peak force (unit increase): IRR 1.3 (1.1–1.6)	Age; sex; BMI; predisposing diseases (0 vs. ≥1)	High (17/20)
Coggon et al. 2013 [53]	CC	GB	*n* = 475 patients; *n* = 799 controls	Abnormal NCS + symptoms (duration ≥1 month)	Repeated movements of wrist >4 h/day; repeated bending of elbow >1 h/day; keyboard/ mouse use >4 h/day; vibration >1 h/day	Repeated movements: OR 1.5 (1.1–1.9); vibration: OR 2.4 (1.6–3.8)	Age; sex; BMI; ethnicity; smoking; other diseases, somatic symptoms; mental health; psychosocial factors	Moderate (9/20)
Goodson et al. 2014 [52]	CC	US	*n* = 87 patients; *n* = 74 controls	Abnormal NCS + symptoms	Repetition; force; repetition + force combined; vibration; total occupational exposure	Repetition: OR 1.8 (1.5–2.2)	Age, BMI, job satisfaction, vigorous exercise; exercise strain; physical activities	Moderate (12/20)

*Abbreviations: ACGIH* American Conference of Governmental Industrial Hygienists, *AL* Action limit, *BMI* Body mass index, *CC* Case control, *CS* Cross-sectional, *HAL* Hand activity level, *HR* Hazard ratio, *IRR* Incidence rate ratio, *MSD* Musculoskeletal disorders, *NCS* Nerve conduction studies, O*NET Occupational Information Network, *OR* Odds ratio, *SI* Strain index score (overall force rating, efforts/min., duration in exertion (%), typical hand/wrist postures; speed of work (h/day)), *TLR* Threshold limit ratio ((Force)/((−0.78)x(HAL) + 7.78)), *TLV* Threshold limit value.

^a^Workers in hospital from central and sterile supply, laboratory, pharmacy, engineering, surgical, kitchen, laundry and administrative support

^b^Exertion per minute were counted from videotape (<10; 10–15; ≥15); percent of time in (forceful) exertion (0–20; 20–60; >60 %)

^c^Force match peak (by dynamometer) represents peak force of job as percent in maximum voluntary contraction MVC/10 (<20 %; 20–70 %; ≥70 %)

^d^Flexion/extension (percent of time spend in range of motion (ROM: 0–20 %; 21–40 %; >40 %)

### Repetition

Seven SRs (two grade B, three grade C and two grade D) examined repetition as a risk factor for CTS (Table 3). On the basis of the highest-quality study available, there is a significant association between repetition and CTS. This association is maintained when only studies that used a conservative CTS case definition [41] are considered. A SR of good quality (grade B) showed that five out of eight studies found a positive association with CTS. The authors concluded that cycles times of <10 s were more harmful than cycles times of <30 s, or when the same movements were performed in >50 % of working time [35]. Another meta-analysis also confirmed this association, though this had not been demonstrated for longitudinal studies [31]. A meta-regression analysis by Abbas et al. [21] showed that country, study population, repetition and force were significant predictors of CTS. Sulsky et al. [44] confirmed that there is consistent evidence for a weak positive relationship between CTS and repetition. Palmer et al. [22] also found that there is an increased risk of CTS from highly repetitive flexion and extension of the wrist. Using the Bradford-Hill criteria for causality, Lozano-Calderón et al. [46] found only slight evidence for a causal relationship between repetition and CTS (Bradford-Hill score: 6.5 of 21 points).

All of the included primary studies confirm that there is a positive association between repetition and CTS (Table 4). The baseline results of Burt et al. [48] show an interaction between BMI and the frequency of exertion per minute (≥5 % of the maximal voluntary contraction). High frequency of exertion (≥15 times/min.) resulted in a three-fold higher probability of CTS in the obese (BMI ≥30). Obesity doubled the odds for CTS among those with frequent exertion per minute. Furthermore, a significant association between HAL and CTS was observed for men but not for women (OR 1.4, 95 % CI 1.05–1.81). Bonfiglioli et al. [50] found that HAL was an independent predictor of CTS (IRR 1.4, 95 % CI 1.19–1.57). According to another study with JEM, occupations requiring frequent repetitive motion were significantly associated with CTS, after adjustment for BMI, age and gender. This applied both to the most recent job and to employment duration time-weighted exposures [51]. Two other longitudinal studies showed that repetition in combination with forceful exertion favours CTS [47, 49]. In both case control studies, CTS patients more often reported that they performed repetitive tasks at work than did control groups [52, 53].

Two SRs (grade B) of good quality confirm an association with repetition. Other SRs (*n* = 5) of lower quality do not provide any contradictory results. The findings of all primary studies also confirm this association. Therefore, the quality of evidence for an association between repetition and CTS was upgraded to a high level.

### Forceful exertion

Seven SRs (two grade B, three grade C and two grade D) examined the association between force and CTS (Table 3). The two current meta-analyses show that force is positively associated with CTS [31, 41]. However, the study results exhibited significant heterogeneity (*I*^*2*^ between 84 and 94). Barcenilla et al. [41] showed that most of the heterogeneity could be explained by factors such as CTS case definition, bias risk and the country of the study. The SR conducted by van Rijn et al. [35] confirmed that there is a positive association with CTS in three of seven original studies (consistency: 43 %). Sulsky et al. [44] reported a weak positive association of questionable validity (two of three studies) and Lozano-Calderón et al. [46] found a weak causal relationship between high force and CTS (Bradford-Hill score: 4.5 of 21 points).

Current primary studies confirm that there is a positive association between force and CTS. At baseline, Burt et al. [48] measured the peak force as the matching value in pounds by dynamometer and expressed as percent of maximum voluntary contraction (% MVC). Workers exposed to a peak force of ≥70 % had a 2.7-fold higher chance of CTS than those with lower levels of peak force (<20 %). Similarly, subjective rating of perceived peak exertion (RPE) on a Borg scale was also positively correlated with a higher probability of CTS (OR 1.14, 95 % CI 1.01–1.29). The longitudinal results show that the percent of time working in forceful exertion had a linear association with CTS. When forceful exertion accounted for more than 20 % of the working time, the risk was increased three-fold; from ≥60 % of the time, the risk was increased 20-fold. Obesity was a significant confounder; when included in the final model, the estimate for forceful exertion increased by 15 % [47]. Garg et al. [49] determined the frequency, intensity and duration of forceful exertion and used this to calculate a Strain Index (SI). At high SI values, the risk of CTS was increased 2.5-fold. Bonfiglioli et al. [50] found that the peak force was a significant predictor of CTS (IRR 1.3, 95 % CI 1.08–1.59). Analysis with JEM confirmed that there is a significant association between forceful motions (dynamic and static strength) at work and the risk of CTS.

Two SRs (grade B) of good quality show a positive association between force and CTS. Other SRs of lower quality did not provide contradictory results. Four primary studies of high quality confirm the positive association. Thus, the quality of evidence for an association between force and CTS was upgraded to a high level.

### Combined exposures (repetition and force)

Two meta-analyses and two SRs (grade B and C, respectively) examined the relationship between CTS and combined exposure patterns (Table 3). Applying the criteria of the National Institute of Occupational Science and Health (NIOSH), Barcenilla et al. [41] found that the risk was doubled, although significant heterogeneity was demonstrated. Subgroup analysis of the studies with a conservative CTS case definition gave borderline significance for a positive association (OR 1.9, 95 % CI 0.99–3.5). In their analyses, Spahn et al. [31] included studies that recorded combined exposure with the ACGIH TLV for HAL score. The authors showed that exposures above TLV were associated with significant increases in the incidence and prevalence of CTS. Two other SRs showed that highly repetitive activities involving forceful exertion increased the risk for CTS in comparison to low exposure [22, 35].

**Fig. 3:**
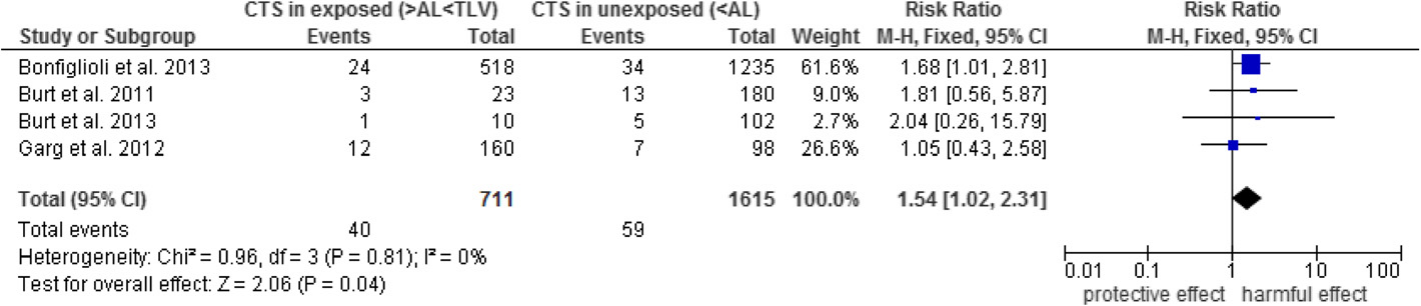
Forest plot of TLV for HAL – below AL versus between AL and the TLV. Outcome: CTS. Abbreviations: AL, action limit; CI, confidence interval; CTS, carpal tunnel syndrome; HAL, hand activity level; TLV, threshold limit value

**Fig. 4:**
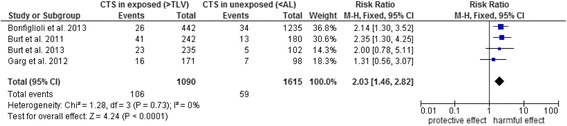
Forest plot of TLV for HAL – below AL versus TLV and above. Outcome: CTS. Abbreviations: AL, action limit; CI, confidence interval; CTS, carpal tunnel syndrome; HAL, hand activity level; TLV, threshold limit value

Five current primary studies examined combined exposures (Table 4); four of these had measured the ACGIH TLV for HAL score and were included in the meta-analysis (Figs. 3 and 4) [47–50]. The analysis shows that the risk for CTS is increased at a moderate HAL for TLV (RR 1.5, 95 % CI 1.02–2.31). Values at or above TLV doubled the risk for CTS (RR 2.0, 95 % CI 1.46–2.82). As the studies are homogenous (*I*^*2*^ = 0), no other stratified analyses were performed.

Two SRs (grade B) of high quality with no conflicting results from other SRs support an association between combined exposures and CTS. Findings from four primary studies (meta-analysis) confirm this association. The quality of evidence for an association between combined exposures such as repetition and force was upgraded to a high level.

### Vibration

Vibration as risk factor was studied in six SRs (two grade B, three grade C and one grade D). Two current meta-analyses demonstrate an association between exposure to hand-held vibratory tools and CTS. These results were based on studies with cross-sectional and case control designs [31, 41]. Van Rijn et al. [35] identified three of five original studies (consistency: 60 %) which observed a significant association between vibration and CTS. Sulsky et al. [44] only included a single high-quality study that demonstrated this association. They concluded that the evidence was insufficient. Palmer et al. [22] included six studies in their descriptive analysis and concluded that exposure to hand-held vibratory tools increases the risk of CTS, particularly when tool use is prolonged (>10 years) and/or intensive (>6 h/day). Lozano-Calderón et al. [46] considered that the exposure was a plausible, but debatable risk factor. The Bradford-Hill score was 6.3 of 21 points, which indicates a weak causal relationship.

Five current primary studies examined exposure to vibration, by asking or observing whether employees worked with vibratory tools; no measurements of frequency or intensity (e.g., acceleration) were collected (Table 4) [47, 48, 50–53]. Except for one case control study, vibration was not associated with CTS [53].

Two qualitatively good SRs (grade B) have established an association between vibration and CTS [35, 41]. However, in current high-quality primary studies, vibration was not an independent strong predictor of CTS. Thus, the quality of evidence for an association between vibration and CTS may be classified as moderate.

### Wrist posture

Seven SRs have examined the effect of extreme wrist flexion and extension on CTS (Table 3). Barcenilla et al. [41] used the NIOSH criteria and found a positive association (OR 2.7, 95 % CI 1.3–5.5). However, there was significant evidence of heterogeneity, due to differences in CTS case definition and bias risk. In contrast, no association was demonstrated in studies with a conservative CTS case definition (OR 4.7, 95 % CI 0.4–53.3). In the meta-analysis conducted by You et al. [43], exposure was defined as wrist deviation in extension or flexion from neutral wrist posture or duration of time in such postures. They found a two-fold risk of CTS among those who frequently work with non-neutral wrist postures during the workday. After consideration of selection and information bias in subgroup analyses, this effect was confirmed (e.g., only cross-sectional study designs or uniform exposure assessment). However, no subgroup analysis was performed of studies with a conservative CTS case definition. The absence of studies with small sample sizes may indicate publication bias. Another meta-analysis demonstrated an association between chronic flexion posture and CTS (OR 1.7, 95 % CI 1.0–2.6). Significant heterogeneity was present; however, no further subgroup analyses were conducted to resolve heterogeneity [31]. Other SRs found that intensive flexion and extension of the wrist for more than 2 h/day was associated with CTS, particularly in combination with repetition and forceful exertion. Some of the listed studies indicate that there may be a dose-response relationship for the number of hours in these hand positions [22, 35]. In accordance with the Bradford-Hill score, the causality for repetitive hand flexion (5.4 of 21 points) and extension (3.6 of 21 points) was classified as low, with a relatively low consistency (about 40 %) [46].

Some inconsistent results between grade B and C reviews were observed for an association between exposure to non-neutral wrist postures and CTS. Current primary studies did not provide further evidence regarding this relationship. We therefore classified the quality of evidence for an association between non-neutral wrist postures and CTS as low.

### Computer use

Two SRs (one grade B and one grade C) which exclusively examined the association between computer use and CTS conclude that there is no evidence for a positive association between computer use and CTS [42, 45]. The results of the meta-analysis demonstrate no association between computer use and CTS (OR 1.7, 95 % CI 0.8–3.6). Moreover, there is no statistically significant association if only the keyboard or mouse use is considered [42]. Other SRs that considered computer use and CTS also failed to find an association (Table 3) [22, 31, 35, 44].

One meta-analysis of good quality (grade B) showed that the epidemiological evidence for a positive association between computer use and CTS is insufficient. Other SRs of lower quality support this finding. Current primary studies do not provide further evidence on this relationship. Thus, the quality of evidence regarding insufficient association between computer use and CTS was considered moderate.

## Discussion

From the epidemiological perspective, this overview and update of the current primary literature confirm that CTS is associated with biomechanical risk factors. High quality of evidence could be established for an association between risk factors such as repetition, forceful exertion, combined exposures and CTS. SRs provide moderate quality of evidence for vibration and low quality of evidence for non-neutral wrist postures. Moderate quality of evidence has been established for an insufficient association between computer use and CTS. Moreover, it has been demonstrated that there is a dose-response relationship between cumulative occupational exposure to force and repetition. A significant increase in risk of CTS was already observed for workers with exposures between AL and the TLV. A further increase in risk was observed for workers with exposures above the TLV.

### Consistency of the results

The SRs on the biomechanical factors repetition, forceful exertion and vibration show high consistency and this is confirmed when different occupational groups, methods of measurement and CTS case definitions are considered.

The study results exhibit weak consistency with respect to non-neutral wrist postures and CTS. According to the review on work-relatedness of biomechanical factors, published by the National Institute of Occupational Science and Health (NIOSH) [10], there was insufficient epidemiological evidence that non-neutral wrist postures are an independent risk factor for CTS. However, a causal association has been established in combination with other biomechanical risk factors [10]. Although three current meta-analyses found a two-fold increased risk of CTS, the results are questionable [31, 41, 43]. When considering only studies with a conservative CTS case definition, no significant effect could be found [41]. In the meta-analysis by Spahn et al. [31] an evident heterogeneity was found; however, no further sensitivity analyses were performed to resolve it. One possible explanation may be the relatively small number of primary studies that examine non-neutral wrist postures as an independent risk factor. Moreover, only a few studies (2 out of 8) employed objective methods of measurement, which could possibly lead to an overestimation of the effect (OR 1.4 versus 3.0) [43]. To deduce reliable exposure levels for flexion and extension, epidemiological studies are required with valid and consistent results for duration and frequency. On the other hand, experimental and clinical studies have found an association between non-neutral wrist postures and CTS. Extreme chronic flexion postures and hyperextension can cause major increases in the carpal tunnel pressure, leading to compression of the N. medianus against the transverse carpal ligament. This can impair the function of the N. medianus – at least in the short term [55].

SRs which studied exposure to computer use came to inconsistent results. An overview of SRs on this association came to the conclusion that there is moderate to high quality of evidence indicating an increased risk of acute pain after intensive use of the mouse or keyboard. However, no evidence was found for the development of specific diseases or chronic pain of the upper extremity [56]. As a complement to Andersen et al.'s [56] overview, we incorporated three additional SRs in our overview, including results from two meta-analyses. These findings provided no further evidence that occupational computer use could lead to relevant increases of CTS [31, 42, 44].

### Strength of association

According to the German Social Security Code (SGB VII, § 9, Sentence 8.2), a disease is predominantly of occupational origin if the risk is increased at least two-fold as a consequence of occupational exposure. This corresponds to an aetiological contribution of at least 50 % in exposed workers. With the exception of computer use, risk was increased at least two-fold with all biomechanical factors. Two meta-analyses found that forceful exertion increased the risk by as much as four-fold [31, 50]. Current primary studies confirm that exposure to highly repetitive and forceful exertion resulted in at least a two-fold risk of CTS.

### Temporality

A causal relationship is plausible when the exposure to the risk factor occurs prior to the onset of the disease. However, most SRs predominantly included studies with case control or cross-sectional designs, as these require relatively low cost and less time. Lozano-Calderón et al. [46] reported that more than 80 % of the examined studies did not describe the aspect of temporality, which implies that there was a lack of prospective studies. The two SRs of good quality only included three to four prospective studies [35, 41]. Therefore, van Rijn et al. [35] argued that the causality of the observed association had not been established beyond doubt. However, we included additional primary studies and found a positive temporal association between exposure and CTS [47, 49–51].

As regards the duration of exposure, a pooled analysis of six prospective studies (*n* = 3515; follow-up: 7 years) found that employees who had been working for less than 3.5 years in their current job exhibited a higher incidence rate than those who had had a comparable job for a longer period (HR 3.08, 95 % CI 1.6–6.1) [57]. Another prospective study found that occupations with high repetition and high forceful exertion were associated with increased incidence of CTS after a short occupational exposure of at least 6 months [51]. An exposure period of up to three years can be sufficient to develop occupational CTS. However, the current studies do not allow the conclusion that longer exposure is an argument against a causal association.

### Dose–response relationship

On the basis of the SRs, it was not possible to reach a reliable conclusion about a dose-response relationship. By using the ACGIH limits, we demonstrated a statistically significant trend for TLV for HAL and CTS [47–50]. A multiplicative effect is probable, as the risk is greater when two exposure factors are combined [50]. Earlier studies also observed a steady increase in prevalence and incidence of CTS in connection with combined biomechanical loads [58–60]. A current analysis with pooled original data from six prospective studies (mainly from the US) did find a significant increase in risk for exposures above the AL (HR 1.7, 95 % CI 1.2–2.5); however, there was no further increase in risk for those with exposures above the TLV (HR 1.5, 95 % CI 1.0–2.1) [61]. In our meta-analysis we also included a large cohort study on CTS (OCTOPUS cohort) from Italy. Overall, this study attained greater weight than other studies in the analysis and showed a steady increase in risk (Figs. 3 and 4) [50]. The authors conclude, that the current ACGIH limits are not adequate as they might not be sufficiently protective and should therefore be revised [50, 61]. The risk of CTS also increases significantly with an increased amount of time spent in forceful exertion. Burt et al. assume that “force may be the primary job exposure risk factor for CTS” and thus, “a reduction in the amount of time spent in forceful exertion and the intensity of the required force of job tasks may reduce the occurrence of CTS” [47]. It should be mentioned that in the meta-analysis on the ACGIH methods, studies based on observational and direct measurements of force were combined. In the study by Bonfiglioli et al. [50], an experienced ergonomist evaluated the peak force by observing the subjects during their work shifts. The other studies assessed the peak force by using a dynamometer [47–49]. The latter approach is objective, while the other approach depends on the judgement of the ergonomist. However, the observational method does not imply any inference with the job tasks and does not depend on the workers’ behaviour.

### Role of confounders

In general, CTS is caused by multiple risk factors. In the general population, CTS is mainly caused by individual risk factors, such as age, gender, obesity and anthropometrics of the hand or other diseases, such as arthritis or diabetes mellitus [62–65]. In contrast to occupational factors, it is comparatively easy to assess individual factors objectively. Nevertheless, a detailed quantitative assessment of the exposure is a major challenge for epidemiological studies, as this must include the frequency, duration and intensity for each activity performed. According to the review based on the Bradford-Hill criteria for causality, the quality and strength of the evidence for a causal association between biological risk factors and CTS were significantly greater than for the occupational risk factors [46]. The authors concluded that, at least at that time, the scientific evidence for occupational risk factors was inadequate and that CTS was largely caused by structural, genetic or biological factors. However, current primary studies with rigorous methods allow the conclusion that occupational risk factors play an important role in the aetiology of CTS. The results of Burt et al. [47, 48] prove that high long-term forceful exertion and repetition are significant independent risk factors for CTS. Obesity (BMI ≥30) is an important confounder. Persons with both individual risk factors such as obesity, as well as occupational risk factors such as high physical demands, have a markedly higher risk of CTS than those with only a single risk factor [48]. It is assumed that the N. medianus is impaired by the fat tissue and/or the swelling in the carpal tunnel [66]. Garg et al. [49] noted that the aetiology of CTS is complex and multifactorial. The combination of repetition and forceful exertion, as measured with the ACGIH TLV for HAL and the Strain Index, was associated with an increased risk of CTS. This was retained after adjustment for essential confounders, such as age, BMI, comorbidities (rheumatoid arthritis and other musculoskeletal diseases of the upper extremities), psychosocial factors and hobbies (gardening). Another prospective study with similar methods also found a significantly increased risk of CTS, after adjustment for known confounders [50].

### Limitations of the primary studies in the SRs

In general, there was considerable heterogeneity between the studies with respect to the assessment of the biomechanical risk factors and the outcome definition. Meta-regression analysis showed that the heterogeneity could be significantly explained by differences in the CTS case definitions, study designs, bias scores and country of study [41]. Differences in CTS diagnostic testing was often mentioned as a limitation in the SRs. Studies with less stringent criteria (e.g., only recording of symptoms) more often found an association, as well as higher values for prevalence and incidence, than did studies with a conservative case definition [46]. Possible consequences might be excessive diagnosis or misclassification of CTS in epidemiological studies [35, 41, 45, 46]. This misclassification is probably differential, as exposed employees may more often suffer symptoms in their hands or fingers than non-exposed participants in the control groups. The combination of a positive nerve conduction test and symptoms or clinical signs gave the most precise results [67]. Barcenilla et al. [41] recommended that a conservative definition of CTS cases should be used in future epidemiological studies. Firstly, this is also used in clinical practice and, secondly, this can probably serve to eliminate a large degree of the studies’ heterogeneity.

There has also been criticism that only a few primary studies used objective or direct measurement procedures to assess exposure [22, 35, 41, 43, 46]. For example, van Rijn et al. [35] indicated that the majority of the included studies (66 %) used self-response to quantify the intensity, frequency or duration of exposures. Moreover, different definitions and exposure limits were applied.

The lack of adequate statistical power is another limitation and is evident in some studies as wide confidence intervals. Abbas et al. [21] remarked that less rigorous studies tend to employ higher but less precise risk estimates. As the use of a conservative CTS case definition is accompanied by lower prevalence and incidence rates, this must be borne in mind when calculating sample sizes for intended prospective studies.

By including primary studies with a conservative CTS case definition, with objective or direct measurements of exposure and appropriate statistical power, heterogeneity could be avoided in the present meta-analysis and a significant association could be demonstrated between occupational biomechanical risk factors and CTS.

### Validity of the included SRs and primary studies

Ultimately, the validity of an overview depends on the validity of the included SRs and primary studies, as well as the processes used for selection and evaluation. One important limitation is that the information we extracted from the SRs has already been filtered and processed by other authors. The reliability of this overview could be greatly influenced by any bias in the review process or by methodological weaknesses in the primary studies included. In an effort to minimise the risk of bias, we prepared this overview in accordance with the PEROSH criteria for SRs and the MOOSE checklist for primary studies. In addition, we excluded narrative reviews, editorials and commentaries a priori.

The methodological validity of the SRs was determined with the AMSTAR-R instrument, which has been found to give a reliable assessment of SRs [32]. Even though the study objectives and outcome were relatively homogenous, the included SRs were of heterogeneous quality. This could be due to differences in the selection and review process, as well as assessment strategy (e.g., study quality and evidence grading). We did not identify any SRs of very good quality (grade A). Only three SRs exhibited good quality (grade B). In general, SRs published within the last five years exhibited higher AMSTAR-R scores which indicates an improvement in the implementation of recommendations for reporting SRs. With one exception [46], the agreement between the reviewers was good to very good (kappa: >0.5). However, items with ambiguous possibilities of interpretation showed a low level of agreement (e.g., Q7: “Was the scientific quality of the included studies assessed and documented?” Q8: “Was the scientific quality used appropriately in formulation conclusions?”). Depending on the research question, SRs of observational studies often used different standards for quality assessment. Although the search strategies or the presentation of the results in the included SRs were relatively consistent, there were considerable differences in the evaluation and interpretation of study quality. There are several checklists and indices for epidemiological studies, particularly observational studies. However, they may differ considerably and do not always fit the planned research question. For this reason, authors sometimes have recourse to a variety of instruments, some of which have not been validated.

In our investigation, the criteria for evaluating the validity of current primary studies were based on publications about similar research questions [35, 36]. This instrument has not been validated, but it covered all essential requirements and was therefore suitable for our purposes. The agreement between the two reviewers was very high: there were no differences in four studies and differences of one to two points in three studies (see Additional file 4). It must be stressed that current primary studies have considered the frequently cited limitations in planning study design. Standardised and objective procedures for testing and measurement are now more frequently used. Statistical power has increased by using larger groups and the period of observation has increased, so that it is now possible to draw conclusions about the temporal link between occupational exposure and outcome.

### Quality of evidence

Establishing the quality of evidence for the risk factors employed a qualitative rather than a formal approach and considered the study validity and consistency of the results from SRs and current primary studies. As shown in a descriptive literature analysis of the methodological implementation of overviews, many authors used the GRADE approach to evaluate the quality of evidence [68]. However, according to Pieper et al. [37], this approach should not be directly transferred to overviews, as some criteria are only suitable for the evaluation of evidence on the basis of primary studies. Overlapping between the study pools is another limitation. In our overview, the overlap rate had the high CCA value of 13.3, i.e., the same references were given in two or more SRs. This led to evidence being counted twice and can considerably restrict its validity. There are currently no formal standardised criteria for this form of summarising evidence. We therefore used a qualitative approach and orientated our study largely towards overviews on musculoskeletal symptoms and diseases, which are also based on epidemiological studies [38, 39, 56]. The postulated core criteria were the validity of the SRs and the consistency of the results. For example, Lakke et al. [39] also considered the validity and consistency of the included cohort studies. Andersen et al. [56] estimated the probability that the observed association is causal. None of these studies carried out an update of current primary literature, so that they were solely dependent on the results of the SRs. Pieper et al. [69] pointed out that the up-to-datedness of the included SRs may be a limitation since new studies may appear during the period between the last literature search and the date of publication. Authors should therefore examine whether including more recent studies may change the conclusion of the review. For this reason, we also included the results of current primary studies in our assessment of the quality of evidence. If there were positive consistent results for biomechanical factors from studies of very high validity, then the quality of the evidence was upgraded. The classification of the degree of evidence into high, moderate, low and poor is not a standardised procedure. This should be borne in mind in the interpretation of the results.

### Search and selection process

As we used a validated sensitive search string for aetiological studies, we assume that we had a high detection rate for relevant SRs and primary studies [30]. The inclusion of six languages led to the identification of a meta-analysis in German [31]. A hand search of references and position papers led to the identification of a report from the Occupational Insurance Association for Safety at Work [44]. The search in the Cochrane database gave no relevant hits. The literature search was limited to a specific time frame, thereby making it impossible to exclude publication bias. Furthermore, due to a small number of studies in the meta-analysis, a statistical test or visual assessment of publication bias was not performed.

In order to ensure comparability, the search for primary studies employed the same sensitive search string, with the exception of the partial string for SRs and meta-analyses. One essential inclusion criterion for primary studies was the use of a conservative CTS case definition; this was the most frequent reason for exclusion.

The results were grouped by the exposure factor and this was found to be expedient, as most of the SRs employed this scheme. We also included computer use as an additional exposure factor, as this was considered to be a risk factor in several SRs.

## Conclusions

Our study of the available epidemiological results leads us to the conclusion that there is high evidence for an increased risk of CTS in activities requiring a high degree of repetition and forceful exertion. The evidence for vibration is moderate. In current primary studies, exposure to vibration is not a strong independent predictor for CTS. We classified the evidence for an association between non-neutral wrist postures and CTS as low, as the results were inconsistent. It may nevertheless be assumed that, in practice, flexion and extension of the wrist mostly occur in combination with other biomechanical factors. There is no further evidence that CTS is caused by working with a computer keyboard or mouse.

With the exception of computer use, the risk was increased at least two-fold and this indicates that occupational mechanical factors are important independent risk factors for CTS. Short periods of exposure are sufficient for occupational CTS to develop. However, SRs and current primary studies do not permit the conclusion that longer exposure times will lead to a reduction in risk of CTS. A dose-effect relationship between combined exposure and CTS has been demonstrated and even moderate exposures (between AL and TLV for HAL) favour the development of CTS.

To avoid heterogeneity, future aetiological studies on CTS in the occupational setting should employ direct objective measurements of individual activities, with a conservative definition of CTS.

When evaluating the association with occupational biomechanical factors, experts are advised to consider competitive factors such as BMI, age, gender or comorbidities, as they might interact with occupational exposures to some extent. Giersiepen and Spallek [70] point out that a clear delineation from a defined occupational disease is often difficult to determine when several conditions in the same body region are present. However, our synthesis of the latest available evidence suggests that CTS should be considered as an occupational disease after certain biomechanical exposures at the workplace.

## Additional files

Additional file 1:
**A proposed reporting checklist for authors, editors and reviewers of meta-analyses of observational studies in epidemiology (MOOSE).** (PDF 24 kb) Additional file 2:
**Search terms and strategy used for MEDLINE, EMBASE, CINAHL Databases.** (PDF 23 kb) Additional file 3:
**Data extraction lists applied separately for systematic reviews and primary studies.** (PDF 18 kb) Additional file 4:
**Quality assessment criteria applied to primary studies and the quality appraisal Table S1.** Quality assessment criteria applied to primary studies; **Table S2.** Quality appraisal of the primary studies. (PDF 29 kb) Additional file 5:
**Appraisal of systematic reviews using AMSTAR-R tool.** (PDF 33 kb)
