# Supplementary material for: Association between work-related biomechanical risk factors and the occurrence of carpal tunnel syndrome: an overview of systematic reviews and a meta-analysis of current research
Source: BMC Musculoskelet Disord. 2015 Sep 1;16:231. doi: 10.1186/s12891-015-0685-0 (PMC4553935; doi:10.1186/s12891-015-0685-0)
Supplement: Additional file 1: — A proposed reporting checklist for authors, editors and reviewers of meta-analyses of observational studies in epidemiology (MOOSE). (PDF 24 kb) [file 12891_2015_685_MOESM1_ESM.pdf]

Additional file 1 – A proposed reporting checklist for authors, editors and reviewers of meta-analyses of observational studies in epidemiology (MOOSE)[a]

Checklist	Yes/no/ n.a.
**Reporting of background**	
1. Problem definition	yes
2. Hypothesis statement	yes
3. Description of study outcome	yes
4. Type of exposure or intervention used	yes
5. Type of study design used	yes
6. Study population	yes
**Reporting of search strategy should include**	
7. Qualification of searchers (e.g. librarians)	yes[1]
8. Search strategy, including time period included in the synthesis and keywords	yes
9. Effort to include all available studies, including contact with authors	yes
10. Databases and registries searched	yes
11. Search software used, name, version, including special features used (e.g. explosion)	yes[2]
12. Use of hand searching (e.g. reference lists of obtained studies)	yes
13. List of citations located and those excluded, including justification	yes
14. Method of addressing articles published in languages other than English	yes
15. Methods of handling abstracts and unpublished studies	no[3]
16. Description of any contact with authors	no[4]
**Reporting of methods should include**	
17. Description of relevance or appropriateness of studies assembled for assessing the hypothesis to be tested	yes
18. Rationale for the selection and coding of data	yes
19. Documentation of how data were classified and coded (e.g. multiple raters, blinding and interrater reliability)	yes
20. Assessment of confounding (e.g. comparability of cases and controls in studies where appropriate)	n.a.
21. Assessment of study quality, including blinding of quality assessors, stratification or regression possible predictors of study results	yes
22. Assessment of heterogeneity	yes
23. Description of statistical methods (e.g. complete description of fixed or random effect models, justification of whether the chosen models account for predictors of study results, dose-response models, or cumulative meta-analysis) in sufficient detail to be replicated	yes

24. Provision of appropriate tables and graphics	yes
**Reporting of results should include**	
25. Graphic summarising individual study estimates and overall estimate	yes
26. Table giving descriptive information for each study included	yes
27. Results of sensitivity testing (e.g. subgroup analysis)	n.a.
28. Indication of statistical uncertainty of findings	n.a.
**Reporting of discussion should include**	
29. Quantitative assessment of bias (e.g. publication bias)	n.a.[5]
30. Justification for exclusion (e.g. exclusion of non-English-language citations)	yes
31. Assessment of quality of the included studies	yes
**Reporting of conclusions should include**	
32. Consideration of alternative explanations for the observed results	yes
33. Generalization of the conclusions (i.e. appropriate for the data presented and within the domain of the literature review)	yes
34. Guidelines for future research	yes
35. Disclosure of funding source	yes

Note. Supplementary materials such as the data extraction form of each systematic review and primary study are available on request. A list of citations located and those excluded, including justification is also available on request.

[a]Stroup DF, Berlin JA, Morton SC, Olkin I, Williamson GD, Rennie D, Moher D, Becker BJ, Sipe TA, Thacker SB: Meta-analysis of observational studies in epidemiology: a proposal for reporting. Meta-analysis Of Observational Studies in Epidemiology (MOOSE) group. JAMA 2000, 283: 2008-2012.

[1]The literature search was compiled and adapted for the three databases MEDLINE, EMBASE and CINAHL with the assistance of an experienced librarian and colleagues with a broad experience in the conduction of systematic reviews.

[2]The results of the literature search were imported and checked for duplicates using the reference management software Mendeley (Version 1.13.0.0).

[3]We did not attempt to identify unpublished data.

[4]Authors were not contacted, as adequate information for the performance of this review was available from publications.

[5]A test for publication bias was not conducted as it may not be accurate for small sample sizes, i.e. small number of studies in the meta-analysis.

Abbreviations. n.a., not applicable; detailed information not provided in the main manuscript.
