# Supplementary material for: Association between work-related biomechanical risk factors and the occurrence of carpal tunnel syndrome: an overview of systematic reviews and a meta-analysis of current research
Source: BMC Musculoskelet Disord. 2015 Sep 1;16:231. doi: 10.1186/s12891-015-0685-0 (PMC4553935; doi:10.1186/s12891-015-0685-0)
Supplement: Additional file 2: — Search terms and strategy used for MEDLINE, EMBASE, CINAHL Databases. (PDF 23 kb) [file 12891_2015_685_MOESM2_ESM.pdf]

Additional file 2 – Search terms and strategy used for MEDLINE, EMBASE, CINAHL databases

Table 1 – Literature search in MEDLINE via PUBMED (Update 27.7.14)

#	Search terms	hits
**#1**	(occupation* OR worker*)	362555
**#2**	(((occupational diseases [MH] OR occupational exposure [MH] OR occupational medicine [MH] OR occupational risk* [TW] OR occupational hazard [TW] OR (industry [MeSH Terms] mortality [SH]) OR occupational group* [TW] OR work-related OR occupational air pollutants [MH] OR working environment [TW])))	173850
**#3**	#1 OR #2	386822
**#4**	((((((exposure [TW]) OR physical load [TW]) OR risk factor* [TW]) OR repetiti* [TW]) OR hand-arm vibration* [TW]) OR force [TW])	1517375
**#5**	#3 OR #4	1788402
**#6**	((((("carpal tunnel syndrome" [MH] OR "carpal tunnel syndrome" [All Fields])) OR ("median nerve neuropathy" [TW] OR "median nerve entrapment [TW]")) OR "nerve compression syndrome" [TW]))	8382
**#7**	#5 AND #6	1655
**#8**	(("meta-analysis as topic"[MH] OR meta-analysis[pt] OR meta-analysis[tiab] OR review[pt] OR review[tiab] NOT (letter[pt] OR editorial[pt] OR comment[pt] NOT ("animals"[MeSH Terms:noexp] NOT "humans"[MeSH Terms])))	2214544
**#9**	#7 AND #8	335
**#10**	("1998/01/01"[PDAT] : "3000"[PDAT])	11159129
**#11**	#9 AND #10	226
**#12**	Filters: Abstracts	222

Table 2 - Literature search in EMBASE via OVID (Update 27.7.14)

#	Search terms	hits
1	(occupation* or worker*).af.	598708
2	exp occupational disease/	132609
3	exp occupational exposure/	66023
4	exp occupational medicine/	61100
5	occupational risk.mp.	2529

6	exp occupational hazard/	14479
7	industry mortality.mp.	13
8	occupational group*.mp.	3053
9	'work-related'.mp.	11855
10	occupational air pollutants.mp.	18
11	working environment.mp.	3371
12	2 or 3 or 4 or 5 or 6 or 7 or 8 or 9 or 10 or 11	255072
13	1 or 12	663920
14	physical load.mp.	1252
15	repetiti*.mp.	94303
16	force.mp.	191343
17	exp exposure/	432491
18	hand-arm vibration.mp.	744
19	risk factor*.mp.	812959
20	14 or 15 or 16 or 17 or 18 or 19	1489407
21	exp carpal tunnel syndrome/ep, et [Epidemiology, Etiology]	1935
22	median nerve neuropathy.mp.	33
23	median nerve entrapment.mp.	147
24	median nerve compression.mp.	483
25	nerve compression syndrome.mp.	215
26	21 or 22 or 23 or 24 or 25	2696
27	review.ab.	919916
28	review.pt.	2058456
29	meta-analysis as topic/	11639
30	meta-analysis.ab.	54831
31	meta-analysis.pt.	0
32	meta-analysis.ti.	38008
33	27 or 28 or 29 or 30 or 31 or 32	2542083
34	letter.pt.	859394
35	editorial.pt.	459424
36	comment.pt.	0
37	34 or 35 or 36	1318818
38	33 not 37	2537782
39	13 or 20	2014011
40	26 and 39	2309
41	38 and 40	426
42	limit 41 to human	410

43	limit 42 to yr="1998 -Current"	262
44	limit 43 to abstracts	228

Table 3 – Literature search in CINAHL via EBSCO (Update 27.7.14)

#	Search terms	hits
S1	TX occupation* or worker*	164434
S2	MH occupational disease	5650
S3	MH occupational exposure	10145
S4	MH occupational medicine	87
S5	TX occupational risk*	2260
S6	TX occupational hazard*	3844
S7	MH industry	7372
S8	MJ mortality	30081
S9	TX occupational group*	1272
S10	TX work-related	3941
S11	TX occupational air pollutants	527
S12	TX working environment.mp.	1336
S13	S2 or S3 or S4 or S5 or S6 or S7 or S8 or S9 or S10 or S11 or S12	59958
S14	S1 or S13	200548
S15	TX exposure	49901
S16	TX physical load	193
S17	TX risk factor*	173132
S18	TX repetiti*	7936
S19	TX hand-arm vibration*	32
S20	TX force	21394
S21	S15 or S16 or S17 or S18 or S19 or S 20	239804
S22	S14 or S21	408060
S23	MH carpal tunnel syndrome	1556
S24	TX carpal tunnel syndrome	1862
S25	TX median nerve neuropathy	19
S26	TX median nerve entrapment	30
S27	TX nerve compression syndrome	51
S28	S23 or S24 or S25 or S26 or S27	1908
S29	S22 and S28	695
S30	PT meta-analysis	0
S31	TI meta-analysis	7191

S32	PT review*	92884
S33	TI review*	77846
S34	S30 or S31 or S32 or S33	165513
S35	S29 and S34	63
S36	S29 and S35 Limiters - Abstract Available; Published Date: 19980101-20141231	45
