# Supplementary material for: Association between work-related biomechanical risk factors and the occurrence of carpal tunnel syndrome: an overview of systematic reviews and a meta-analysis of current research
Source: BMC Musculoskelet Disord. 2015 Sep 1;16:231. doi: 10.1186/s12891-015-0685-0 (PMC4553935; doi:10.1186/s12891-015-0685-0)
Supplement: Additional file 3: — Data extraction lists applied separately for systematic reviews and primary studies. (PDF 18 kb) [file 12891_2015_685_MOESM3_ESM.pdf]

Additional file 3 – Data extraction from systematic reviews and primary studies

Systematic reviews (1998-2014)

1. Author, year
2. Country
3. Study aim(s)
4. Study design
5. Comprehensive literature search and evaluation process (e.g. years, databases, languages, inclusion/exclusion criteria, duplicate study selection)
6. Number of the original studies included in the systematic reviews (number of studies used to assess the degree of overlap between the systematic reviews)
7. Study designs of the included original studies
8. Occupational professions included in the original studies
9. Outcome definition
10. Exposure factors and main results (e.g. effect estimates from meta-analyses)
11. Method used to aggregate the results from original studies (quantitative or qualitative)
12. Methodological quality assessment of the included studies
13. Criteria used for grading the quality of evidence
14. Number of studies which observed a positive significant association between the defined exposure and outcome (consistency of the available evidence)
15. Assessment of publication bias
16. Recognition of scientific quality in formulating conclusions (strengths and limitations)
17. AMSTAR-R score

Primary studies (2011-2014)

1. Author, year
2. Country
3. Study aim(s)
4. Study design
5. Follow-up period (if cohort study)
6. Study population (e.g. occupational profession, gender, age, definition of patients and controls)
7. Participation rate at baseline and at follow-up (if applicable)
8. Outcome definition and assessment (e.g. blinded)

9. Exposure factors definition and assessment (e.g. objective method, blinded)
10. Main results from multivariate analyses (e.g. effect estimates or raw data)
11. Recognition of confounder
12. Method used for data analysis
13. Recognition of strengths and limitations in formulating conclusions
14. Quality score
