# Supplementary material for: Association between work-related biomechanical risk factors and the occurrence of carpal tunnel syndrome: an overview of systematic reviews and a meta-analysis of current research
Source: BMC Musculoskelet Disord. 2015 Sep 1;16:231. doi: 10.1186/s12891-015-0685-0 (PMC4553935; doi:10.1186/s12891-015-0685-0)
Supplement: Additional file 4: — Quality assessment criteria applied to primary studies and the quality appraisal Table S1. Quality assessment criteria applied to primary studies; Table S2. Quality appraisal of the primary studies. (PDF 29 kb) [file 12891_2015_685_MOESM4_ESM.pdf]

Additional file 4 – Quality assessment criteria applied to primary studies and the quality appraisal

Table 1 - Quality assessment criteria applied to primary studies

**Study design and purpose (n = 4 items)**
1. Positive if a specific, clear study aim was described;
2. Positive if the study design was prospective or a retrospective cohort;
3. Positive if the follow-up period of the study was at least one year;
4. Positive if a clear definition of patients and comparators were provided (e.g. exclusion criteria: patients with recurrent CTS or patients who had had carpal tunnel decompression prior to the beginning of the study) and if the patients and comparators were drawn from the same population;
**Study population (n = 4 items)**
5. Positive if the main features of the study population were stated (e.g. distribution by age and gender);
6. Positive if the participation rate of the study population was at least 70% at baseline;
7. Positive if the participation rate of the study population was at least 70% at follow-up (≥ 1 year);
8. Positive if the total number of patients was n ≥ 50;
**Assessment of exposure (n = 6 items)**
9. Positive if the exposure (physical load at work) was clearly defined and used in the analysis;
10. Positive if the data on exposure were collected using standardised methods of acceptable quality;
11. Positive if the exposure was assessed by an independent person and was not based on self-reported exposure;
12. Positive if the data on physical factors during leisure time (e.g. hobbies) were collected and used in the analysis;
13. Positive if the exposure assessment was blinded with respect to the outcome status;
14. Positive if the exposure was assessed at a time prior to the occurrence of the outcome;
**Assessment of outcome (n = 3 items)**
15. Positive if the outcome was clearly defined;
16. Positive if the data on outcome were collected by an independent person using standardised methods of acceptable quality (e.g. not based on self-reports);
17. Positive if the outcome was blinded with respect to the exposure status;
**Data analysis and presentation (n = 3 items)**
18. Positive if the study controlled for confounders;

19. Positive if the statistical method used was appropriate for the outcome studied and for the control of confounders;

20. Positive if risk estimates were presented or when raw data were provided.

Table 2 – Quality appraisal of the primary studies

**Items**	**1**	**2**	**3**	**4**	**5**	**6**	**7**	**8**	**9**	**10**	**11**	**12**	**13**	**14**	**15**	**16**	**17**	**18**	**19**	**20**	**Disagreements (items)**	**Total Score**	**Quality* (max. 20 points)**
Bonfiglioli et al. 2013 [50]	+	+	+	+	+	+	-	+	+	+	+	-	+	+	+	+	-	+	+	+	16	17	high
Burt et al. 2011 [48]	+	-	-	+	+	+	n.a.	+	+	+	+	+	+	n.a.	+	+	+	+	+	+		16	high
Burt et al. 2013 [47]	+	+	+	+	+	+	+	-	+	+	+	+	+	+	+	+	+	+	+	+		19	high
Coggon et al. 2013 [53]	+	-	-	+	+	n.a.	n.a	+	-	-	-	-	-	n.a.	+	+	-	+	+	+		9	moderate
Evanoff et al. 2014 [51]	+	+	+	+	+	n.a.	-	-	+	+	-	-	n.a.	+	+	+	n.a.	+	+	+	10	13	moderate
Garg et al. 2012 [49]	+	+	+	+	+	+	n.a.	-	+	+	+	+	+	+	+	+	+	+	+	+		18	high
Goodson et al. 2014 [52]	+	-	-	+	+	n.a.	n.a.	+	+	+	-	+	-	n.a.	+	+	-	+	+	+	5; 19	12	moderate

Note. Items scored positive (+); negative (-) or n.a., not applicable. *According to the sum of positive scores, the quality is ranked into high (≥14 points), moderate (8-13 points) and low (≤7).
