# Supplementary material for: Association between work-related biomechanical risk factors and the occurrence of carpal tunnel syndrome: an overview of systematic reviews and a meta-analysis of current research
Source: BMC Musculoskelet Disord. 2015 Sep 1;16:231. doi: 10.1186/s12891-015-0685-0 (PMC4553935; doi:10.1186/s12891-015-0685-0)
Supplement: Additional file 5: — Appraisal of systematic reviews using AMSTAR-R tool. (PDF 33 kb) [file 12891_2015_685_MOESM5_ESM.pdf]

Additional file 5 – Appraisal of systematic reviews using AMSTAR-R tool

**AMSTAR-R Item**	**1**		**2**		**3**		**4**		**5**		**6**		**7**		**8**		**9**		**10**		**11**		**Final Score**	**κ**
Rater (AK/ TW)	AK	TW	AK	TW	AK	TW	AK	TW	AK	TW	AK	TW	AK	TW	AK	TW	AK	TW	AK	TW	AK	TW		
You et al. 2014 [43]	3	3	1	1	3	3	2	2	1	1	4	4	2	1 (2)	2	1 (2)	4	4	4	4	2	2	28	0.76
Mediouni et al. 2014 [42]	3	3	2	2	4	4	3 (4)	4	4	4	4	4	1	1	1	1	4	4	3	3	2	2	32	0.87
Barcenilla et al. 2012 [41]	3	3	4	4	4	4	2	2	2	2	4	4	3	4 (3)	4	3 (4)	4	4	4	4	2	2	36	0.69
Spahn et al. 2012 [31]	3	3	2	2	4	4	4	4	1	1	3	4 (3)	1	1	1	1	3	4 (3)	3	3	2	2	27	0.76
Van Rijn et al. 2009 [35]	3	3	4	4	3	3	4	4	1	1	4	4	3	4 (3)	3 (2)	2	1	1	1	1	2	2	29	0.76
Lozano-Calderón et al. 2008 [46]	2	3 (2)	2	2	2 (1)	1	2	2	1	1	2	2	3 (2)	2	3	2 (3)	1	1	1	1	2	2	19	0.38
Thomsen et al. 2008 [45]	3	3	1	1	4	4	2	2	1	1	4	4	3	2 (3)	3	2 (3)	1	1	1	1	3	3	26	0.76
Palmer et al. 2007 [22]	3 (2)	2	4	4	4	4	2	2	1	1	4	4	1	1	1	1	1	1	1	1	3	3	24	0.87
Sulsky et al. 2005 [44]	3	3	4	4	1	1	3 (4)	4	1	1	4	4	3	4 (3)	4 (3)	2 (3)	1	1	1	1	2	1 (2)	27	0.48
Abbas et al. 1998 [21]	2	2	1	1	1	1	2	2	1	1	4	4	1	1	1	1	2	1 (2)	3	3	2	2	20	0.86
Number of mismatch per item	2		0		1		2		0		1		6		6		2		0		1			

Note. Number in brackets after consensus agreement. Numeric AMSTAR-R quality score in grades: A=37-44; B=29-36; C=21-28; D=13-20 points.
Abbreviations: κ Cohen's Kappa Coefficient.
